# Correction of *F8* intron 1 inversion in hemophilia A patient-specific iPSCs by CRISPR/Cas9 mediated gene editing

**DOI:** 10.3389/fgene.2023.1115831

**Published:** 2023-03-09

**Authors:** Zhiqing Hu, Yong Wu, Rou Xiao, Junya Zhao, Yan Chen, Lingqian Wu, Miaojin Zhou, Desheng Liang

**Affiliations:** ^1^ Center for Medical Genetics, School of Life Sciences, Central South University, Changsha, China; ^2^ Shenzhen Baoan Women’s and Children’s Hospital, Jinan University, Shenzhen, China

**Keywords:** hemophilia A, F8 intron 1 inversion, *in situ* gene addition, homology-mediated end joining, hepatocyte-like cells

## Abstract

**Introduction:** Hemophilia A (HA) is the most common genetic bleeding disorder caused by mutations in the *F8* gene encoding coagulation factor VIII (FVIII). As the second predominant pathogenic mutation in hemophilia A severe patients, *F8* Intron one inversion (Inv1) completely splits the *F8* gene into two parts and disrupts the *F8* transcription, resulting in no FVIII protein production. The part which contains exon 2-exon 26 covers 98% of *F8* coding region.

**Methods:** We hypothesized that *in situ* genetic manipulation of *F8* to add a promoter and exon one before the exon two could restore the *F8* expression. The donor plasmid included human alpha 1-antitrypsin (hAAT) promoter, exon one and splicing donor site (SD) based on homology-mediated end joining (HMEJ) strategy was targeted addition in hemophilia A patient-derived induced pluripotent stem cell (HA-iPSCs) using CRISPR/Cas9. The iPSCs were differentiated into hepatocyte-like cells (HPLCs).

**Results:** The hAAT promoter and exon one were targeted addition in HA-iPSCs with a high efficiency of 10.19% *via* HMEJ. The FVIII expression, secretion, and activity were detected in HPLCs derived from gene-targeted iPSCs.

**Discussion:** Thus, we firstly rescued the 140 kb reversion mutation by gene addition of a 975 bp fragment in the HA-iPSCs with Inv1 mutation, providing a promising gene correction strategy for genetic disease with large sequence variants.

## 1 Introduction

Hemophilia A (HA) is an X-linked recessive genetic bleeding disorders with the incidence of one in 5,000 male births (Berntorp et al., 2021; Ragni, 2021). Affected males suffer from spontaneous soft-tissue, muscle and joint bleeding symptoms, the severe patients (coagulation factor VIII (FVIII) activity <1% of the normal value) even experience life-threatening intracranial hemorrhage (Song et al., 2021). HA is caused by the deficiency of functional FVIII, encoded by *F8* gene, which is one of the largest genes spanning 186 kb on Xq28 (Lassalle et al., 2020).

Traditionally, HA is treated by FVIII protein replacement. Owing to the short half-life of FVIII (14–19 h), the HA patients need repeat injections of the FVIII, resulting in huge economic burden on the patients and their families (Batty and Lillicrap, 2019). In recent decades, HA gene therapy was developed and made breakthrough (Perrin et al., 2019; Rodriguez-Merchan et al., 2021; Ozelo et al., 2022). The codon-optimized BDD-*F8* was transduced into hepatocytes using AAV5 vectors and the median FVIII coagulation activity was maintained 20 IU/dL in the patients treated with high dose AAV (6 × 10^13^ vg/kg) in a 3-year follow-up study (Pasi et al., 2020) and the European Commission granted conditional marketing authorization to valoctocogene roxaparvovec gene therapy on 24 August 2022. However, the transduced *F8 via* AAV vector wasn’t integrated into the genome and the *F8* coding sequence (7 kb) far exceeds the packaging capacity of AAV (4.7 kb) (Tornabene and Trapani, 2020; Marrone et al., 2022). Even the B domain deleted *F8* version cannot be easily packaged into the AAV vector. Considering these issues and some HA patients with AAV antibodies (Verdera et al., 2020), gene therapy strategy *via* non-viral system was developed actively. Since the strategy of *in situ* gene repair for HA enables retention of the main *F8* gene regulatory elements, the strategy has been extensively investigated.

*F8* Intron one inversion (Inv1) is the second predominant pathogenic mutation in severe HA patients. In human genome, the reverse repeat of a 1,041-bp sequence within *F8* intron one is located in 140 kb telomeric to the *F8* gene, and this repeat may induce intrachromosomal recombination during male meiosis and cause large inversion (Inv1) (Fahiminiya et al., 2021). This large inversion completely splits the *F8* gene into two parts and disrupts the *F8* transcription, resulting in no FVIII protein production. Notably, the part which contains exon 2-exon 26 covers 98% of *F8* coding region. Thus, we hypothesized that *in situ* genetic manipulation of *F8* to add a promoter and exon 1 (146 bp) before the exon two might represent a therapeutic strategy for restoring the reading frame for all HA patients with *F8* Inv1mutations.

In this study, we performed a targeted addition of human alpha 1-antitrypsin (hAAT) promoter and exon one before the exon two in HA patient-derived induced pluripotent stem cell (HA-iPSCs) using CRISPR/Cas9 and donor plasmid. To achieve a higher integration efficiency, we constructed a donor plasmid with the homologous arms flanking with two same sgRNA4 sites to excise the backbone sequences based on homology-mediated end joining (HMEJ) strategy (Yao et al., 2017; Li et al., 2021; Yuan et al., 2021). Meanwhile, a donor plasmid for classic homologous recombination (HDR) and a donor plasmid for non-homologous end joining (NHEJ) were constructed as control. The integration efficiency in HMEJ group was 10.19%, higher than that in HDR group (6.25%) and NHEJ group (0.99%). The *F8* transcript and FVIII secretion were rescued in the hepatocyte-like cells (HPLCs) derived from gene targeted iPSCs. Our findings provide an *in situ* genetic addition strategy which is promising for the clinical translation in gene therapy for HA involving large sequence variants.

## 2 Results

### 2.1 Characterization of HA-iPSCs

We previously generated an iPSC line (HA-iPSCs) derived from the urine cells of a HA patient with *F8* Inv1 (Hu et al., 2015). Here the HA-iPSCs were identified *via* immunofluorescence. The HA-iPSCs maintained pluripotency according to immunofluorescence. The HA-iPSCs expressed Oct4, Nanog and SSEA-4, while SSEA-1 wasn’t expressed (Supplementary Figure S1A). To further evaluate the pluripotency *in vivo*, HE staining of teratomas was performed and the results showed that teratomas contained ectoderm, endoderm and mesoderm (Supplementary Figure S1B). Meanwhile, the HA-iPSCs maintained a normal karyotype (Supplementary Figure S1C).

### 2.2 Generation of CRISPR/Cas9 and donor template for *in situ* gene addition

Inv1 of *F8* splits the *F8* gene into two parts and disrupts the *F8* transcription, resulting in no FVIII protein production. The part which contains exon 2-exon 26 covers 98% of *F8* coding region. So the Inv1 mutation could be rescued by gene addition of a promoter and the coding sequences of exon 1 (Figure 1). We then designed six single-guide RNAs (sgRNAs) F8-sg1, F8-sg2, F8-sg3, F8-sg4, F8-sg5, and F8-sg6 mapping to target sites in intron 1 (Figure 2A) and constructed and verified the cleavage activity *via* T7 Endonuclease I (T7E1) (Figure 2B). The cleavage frequency of F8-sg4 was 56.53% and was used for targeted addition. The donor plasmids were designed (Figure 2C), constructed and verified with Sanger sequencing (Figure 2D).

**FIGURE 1:**
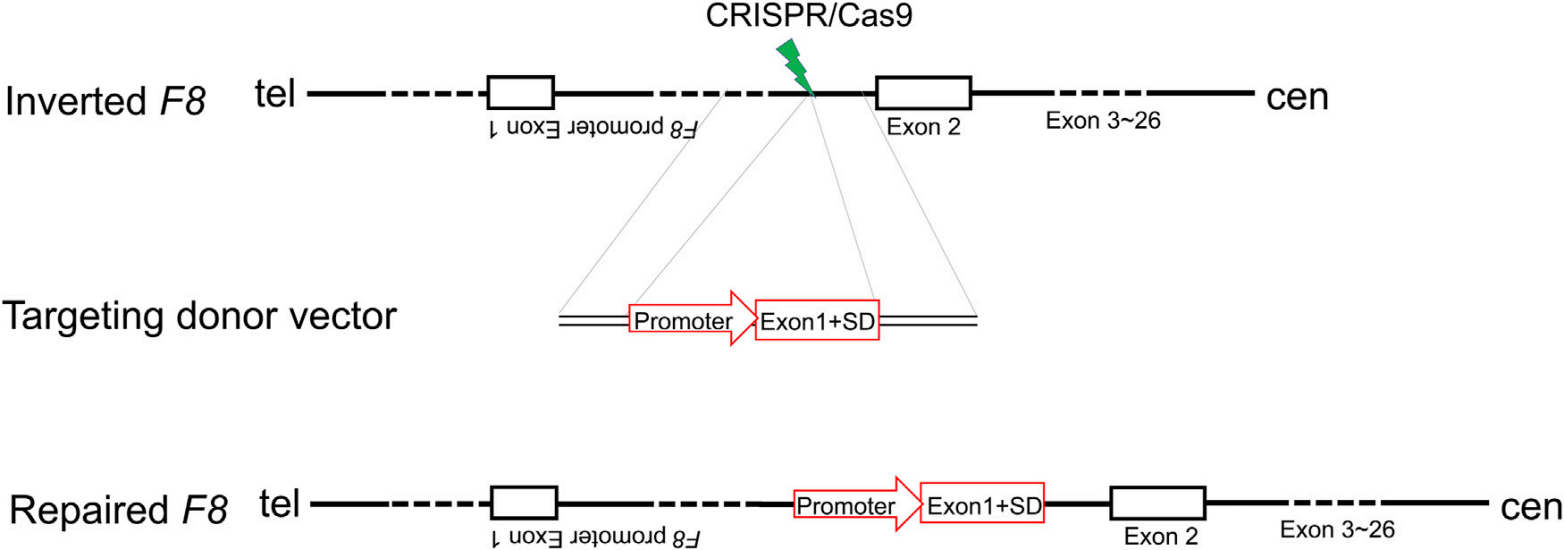
Schematic illustration of *in situ* gene addition strategy for Inv1 of *F8*. SD, splice donor site.

**FIGURE 2:**
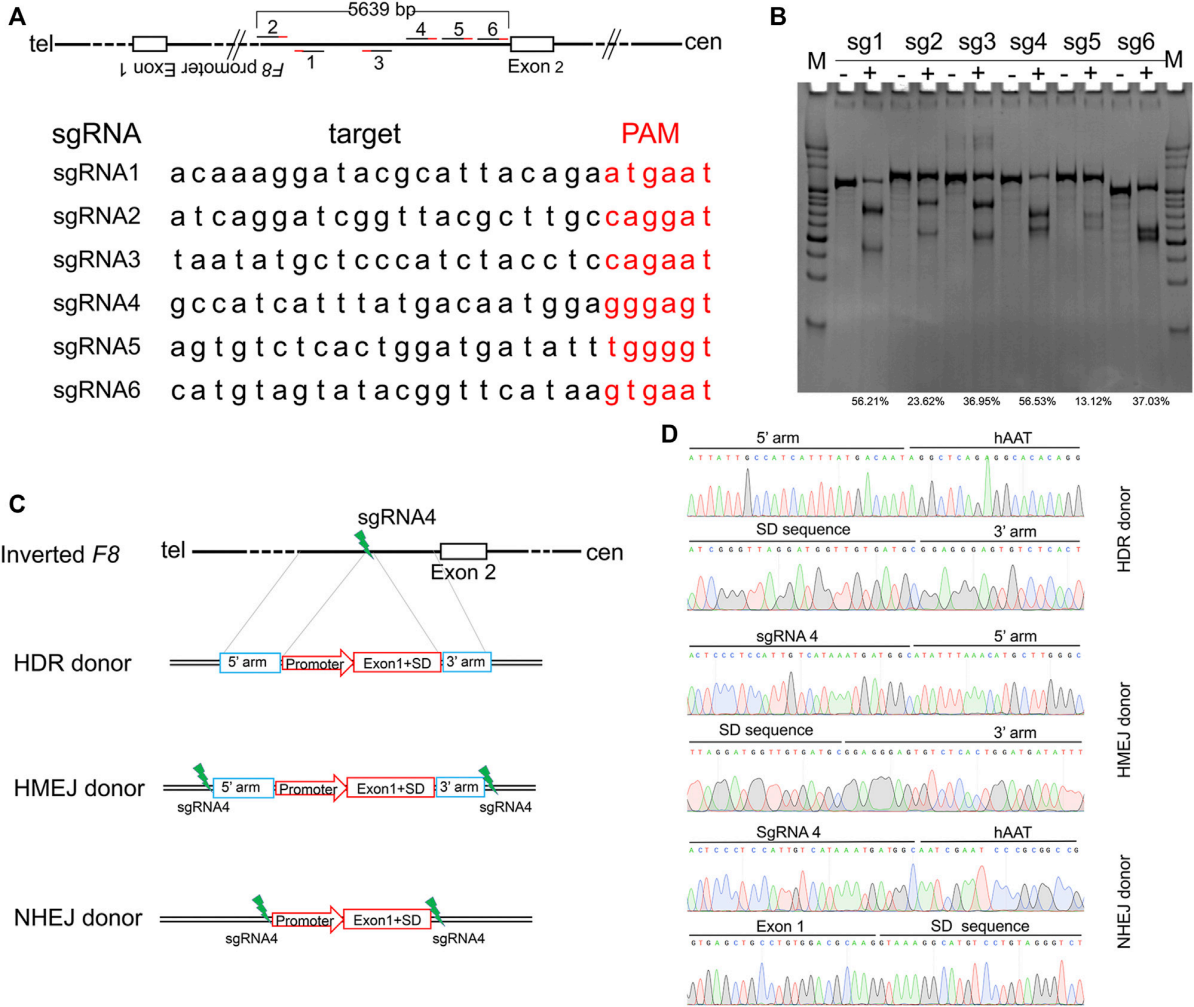
Gene editing components for *in situ* gene addition. **(A)** Schematic illustration of sgRNAs position and the sequences of sgRNAs. The PAM sequences of CRISPR/Cas9 are red-labeled bases. **(B)** T7E1 assay for CRISPR/Cas9 efficiency detection. The percentage of indels was shown. **(C)** Schematic diagrams of the donor template. The donor template includes HDR donor, HMEJ donor and NHEJ donor. 5′ arm, the 5′ homologous arm; 3′ arm, the 3′ homologous arm. **(D)** Sanger sequencing of the donor plasmid.

### 2.3 CRISPR/Cas9 and donor plasmid mediated targeted addition

The HA-iPSCs were nucleofected with the plasmids expressing the CRISPR/Cas9 complex and F8-sg4 along with the donor plasmid F8-NHEJ, F8-HDR, F8-HMEJ, respectively. The single-cell clone was screened using primers across homology arms 5F/R (Figure 3A) and 3F/R (Figure 3B), and the sequences were verified *via* Sanger sequencing (Figure 3C). The targeting efficiency was 10.19% with the donor plasmid F8-HMEJ, higher than that in the F8-HDR group (6.25%) and the F8-NHEJ group (0.99%) (Table 1). Two targeted addition clones (T-26 and T-73) generated from the HA-iPSCs were then used for further research. The primers 5F and 3R were used to detect the purity of the single-cell clone (Figure 3D). The immunofluorescence showed that T-26 and T-73 maintained pluripotency (Figure 3E). The HE staining of teratomas further confirmed the pluripotency *in vivo* (Figure 3F), and T-26 and T-73 maintained a normal karyotype (Figure 3G). To evaluate the off-target effect of CRISPR/Cas9, the potential off-target sites (≤4 mismatches) of F8-sg4 predicted using CHOPCHOP (http://chopchop.cbu.uib.no/) were amplified and sequenced. No off-target indels in the potential off-target sites were observed comparing the sequences in HA-iPSCs with that in T-26 and T-73 (Supplementary Figure S2).

**FIGURE 3:**
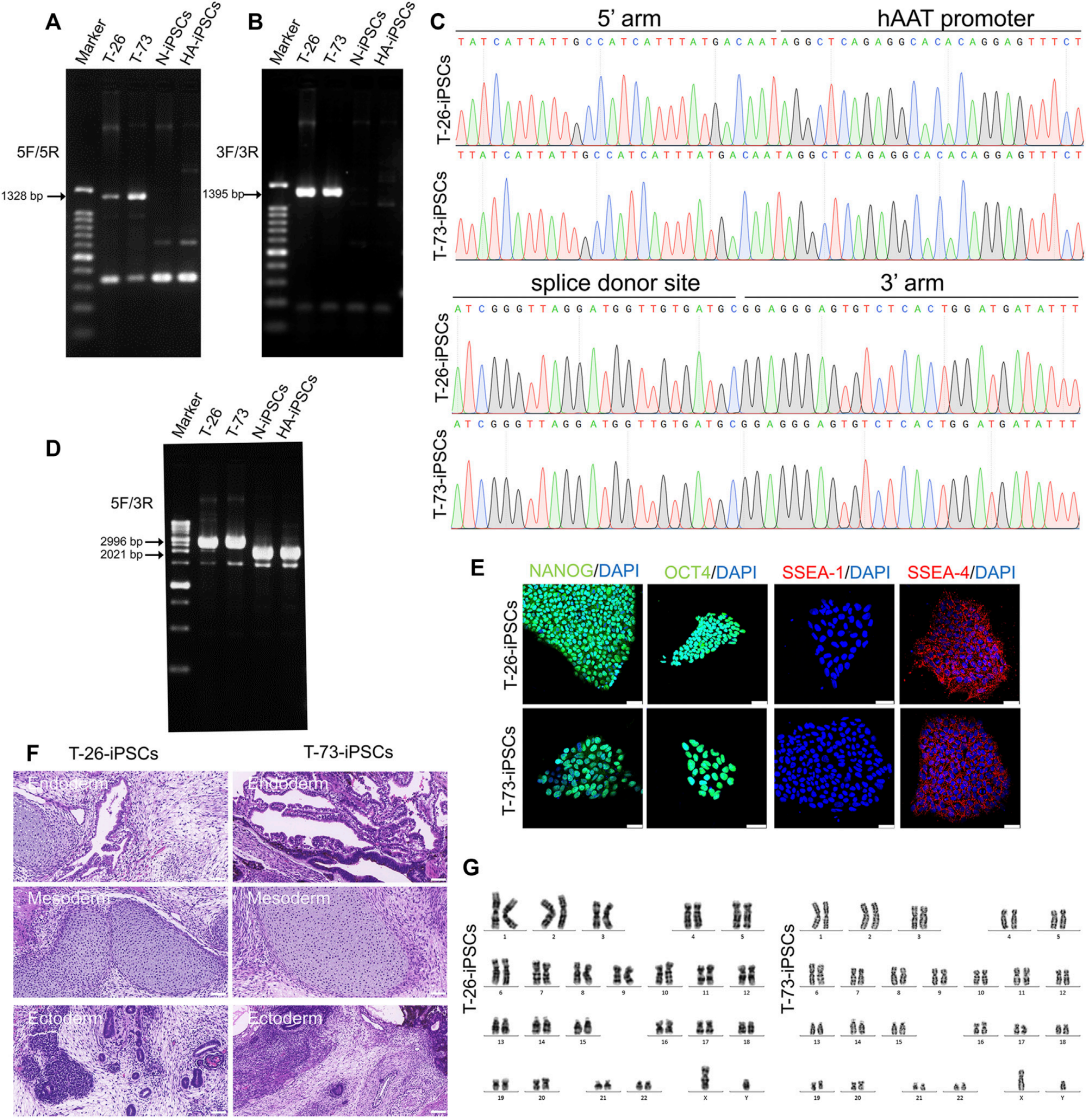
Gene targeting of the HA-iPSCs. **(A)** PCR screening of gene targeted iPSCs using the primers across homology arms 5F/5R. Sizes of the PCR products: T-26, 1,328 bp; T-73, 1,328 bp; No PCR product was obtained for the N-iPSCs and HA-iPSCs. **(B)** PCR screening of gene-targeted iPSCs using the primers across homology arms 3F/3R. Sizes of the PCR products: T-26, 1,395 bp; T-73, 1,395 bp; No PCR product was obtained for the N-iPSCs and HA-iPSCs. **(C)** The PCR products for T-26 and T-73 iPSCs using primer 5F/5R and 3F/3R were sequenced by Sanger sequencing. **(D)** PCR screening of T-26, T-73, HA-iPSCs, and N-iPSCs using primers 5F/3R. PCR products sizes: T-26, 2,996 bp; T-73, 2,996 bp; N-iPSCs, 2021 bp; and HA-iPSCs, 2021 bp. **(E)** Immunofluorescence staining indicated that T-26 and T-73 iPSCs expressed the markers NANOG, OCT4, SSEA-4 but not SSEA-1. DAPI was used for nuclear staining. Scale bar: 50 µm. **(F)** H&E staining of teratomas derived from T-26 and T-73. The teratomas contained three germ layers (ectoderm, mesoderm and endoderm). Scale bar: 200 µm. **(G)** Karyotype of T-26 and T-73 iPSCs.

**TABLE 1:** Summary of the three donor plasmids for gene addition.

Donor plasmid	Clones analyzed	Corrected clones	Targeting efficiency (%)
NHEJ	101	1	0.99
HDR	96	6	6.25
HMEJ	108	11	10.19

Considering a modified hAAT promoter was used in the gene addition, we detected the transcription of the hAAT gene and found that the hAAT gene was transcribed in the iPSCs (Figure 4A). Then the *F8* transcription was detected in T-26 and T-73 *via* reverse transcription PCR (RT-PCR), while no *F8* transcript was detected in HA-iPSCs (Figure 4B). The sequencing results revealed that the promoter and the exon one were successfully inserted into *F8* in T-26 and T-73 with the *F8* transcription restored (Figure 4C), demonstrating the *F8* expression was rescued in the gene targeting group.

**FIGURE 4:**
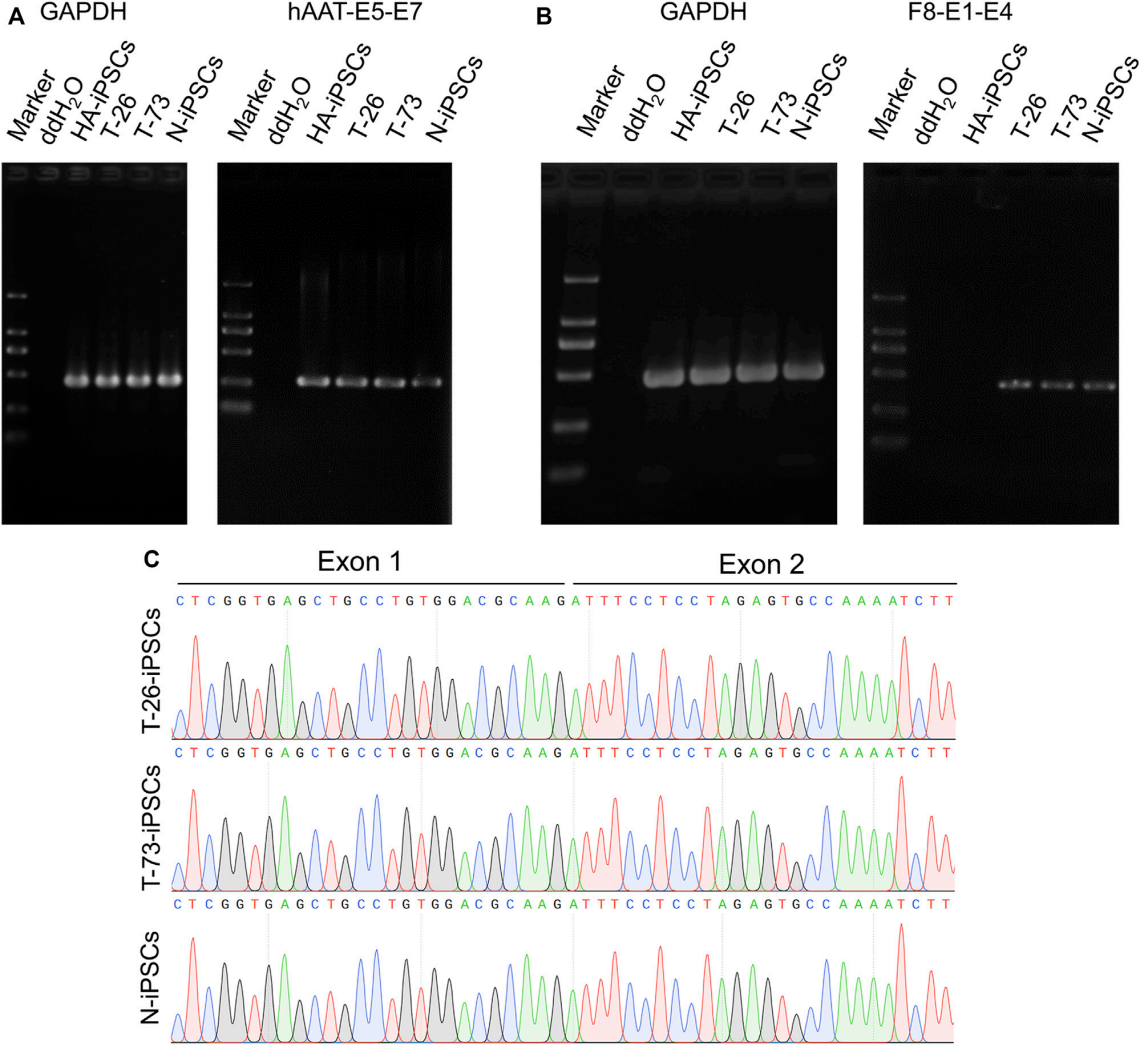
F8 transcription in gene corrected iPSCs. **(A)** RT-PCR analysis of hAAT transcription in iPSCs. The hAAT-E5-E7 using primers targeting exons five and seven of hAAT gene. GAPDH was used as a loading control. **(B)** F8 expression in iPSCs according to RT-PCR. F8-E1-E4 using primers targeting exons one and four of F8. GAPDH was used as a loading control. **(C)** Sanger sequencing of the products of RT-PCR.

### 2.4 Differentiation of targeted iPSCs into hepatocyte-like cells

To evaluate the *F8* expression in hepatocyte, we differentiated the HA-iPSCs, the gene-corrected iPSCs T-26 and T-73, the normal hiPSCs (N-iPSCs) into the hepatocyte-like cells (HA-iHPLCs, T-26-iHPLCs, T-73-iHPLCs, and N-iHPLCs) as the diagram in Figure 5A. During the differentiation, the cells went through four stages. The cell morphology was gradually changed from iPSC clone to epithelioid cell morphology (Figure 5B) and identified the cell marker with immunofluorescence. All cells in the first stage expressed the definitive endoderm cell markers SOX17 and FOXA2; 5 days later, the AFP signal was positive in hepatoblast-like cells; after 5 days of culture, followed by 11 days of culture in Hepatocyte Culture Medium (HCM) contained 20 ng/mL oncostatin M (OsM), the hepatocyte-like cells expressed ALB (Figure 5C). In addition, differentiated HPLCs on Day 25 with characteristic functions of mature hepatocytes could store glycogen and metabolize indocyanine green (ICG) (Figure 5D, E).

**FIGURE 5:**
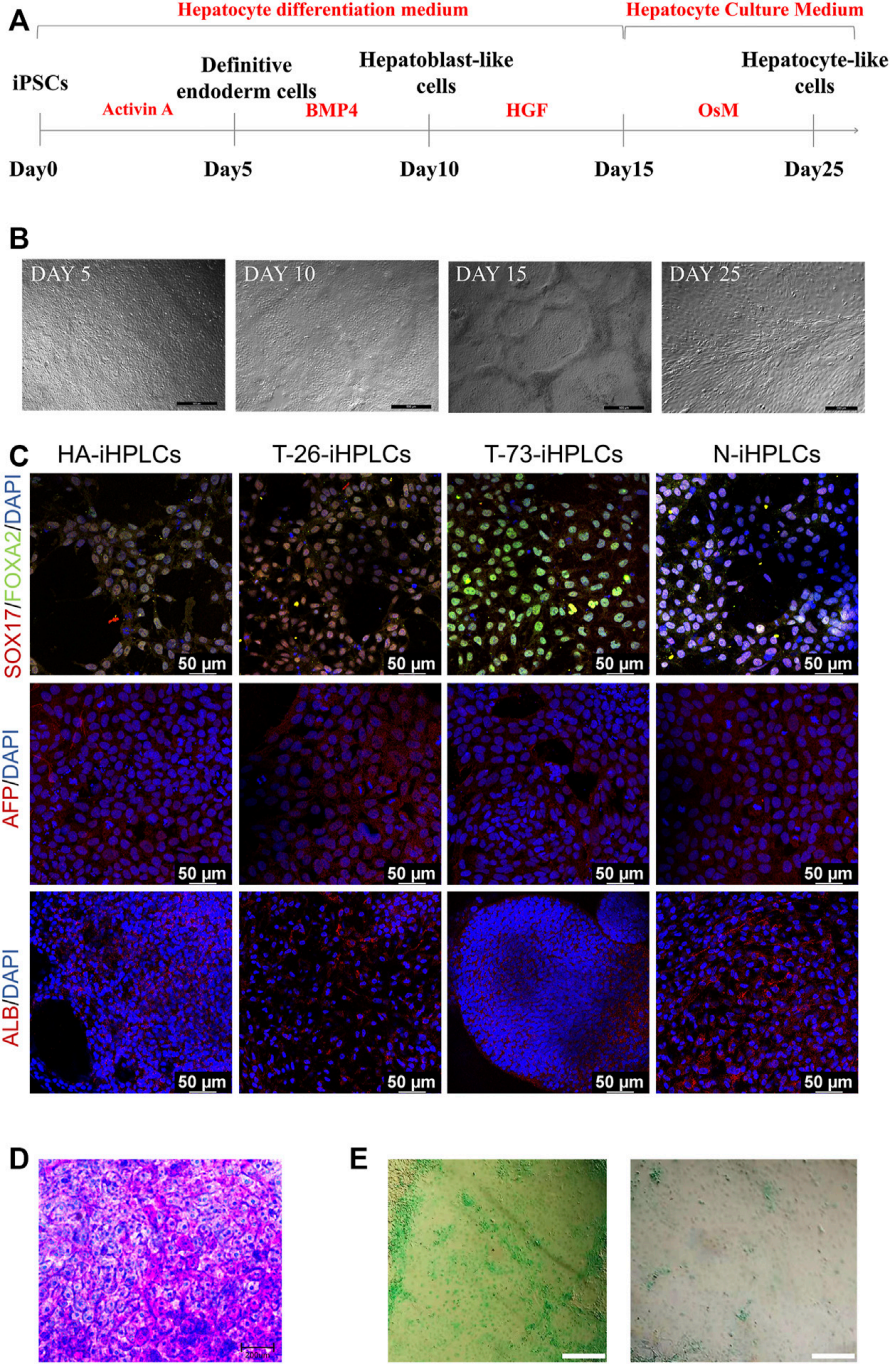
The hepatocyte-like cells derived from iPSCs. **(A)** Schematic diagram of the protocol for differentiation of iPSCs into HPLCs. **(B)** The cell morphology in different stage during differentiation of iPSCs into HPLCs. Scale bar: 200 μm. **(C)** Immunostaining for HPLCs derived from iPSCs at different stages. Immunofluorescence staining of SOX17 (red), FOXA2 (green), AFP (red), and ALB (red). DAPI was used for nuclear staining (blue). Scale bar: 50 µm. **(D)** Periodic acid-Schiff’s (PAS) staining for the mature HPLCs on Day 25. The stained cells showed the ability to store glycogen. Scale bar: 200 µm. **(E)** ICG uptake assay for the mature HPLCs on Day 25. The stained cells indicate the ability to metabolize ICG. Scale bar: 200 µm.

### 2.5 F8 expression in iPSCs derived hepatocyte-like cells

RT-PCR results indicated that the *F8* transcripts were detected in T-26-iHPLCs and T-73-iHPLCs (Figure 6A). The supernatant of HA-iHPLCs, T-26-iHPLCs, T-73-iHPLCs, and N-iHPLCs were collected for FVIII antigen detecting using ELISA. Results showed that human FVIII antigen secreted by T-26-iHPLCs was higher than that in HA-iHPLCs (Figure 6B). And the FVIII coagulation activities in the supernatants from the T-26-iHPLCs were detectable by FVIII activity assay (Figure 6C). More importantly, the FVIII was expressed in T-26-iHPLCs, T-73-iHPLCs *via* immunofluorescence staining, but no FVIII was detected in HA-iHPLCs due to the interrupted *F8* gene (Figure 6D). These results suggest that FVIII expression was restored in gene-corrected iPSCs derived HPLCs.

**FIGURE 6:**
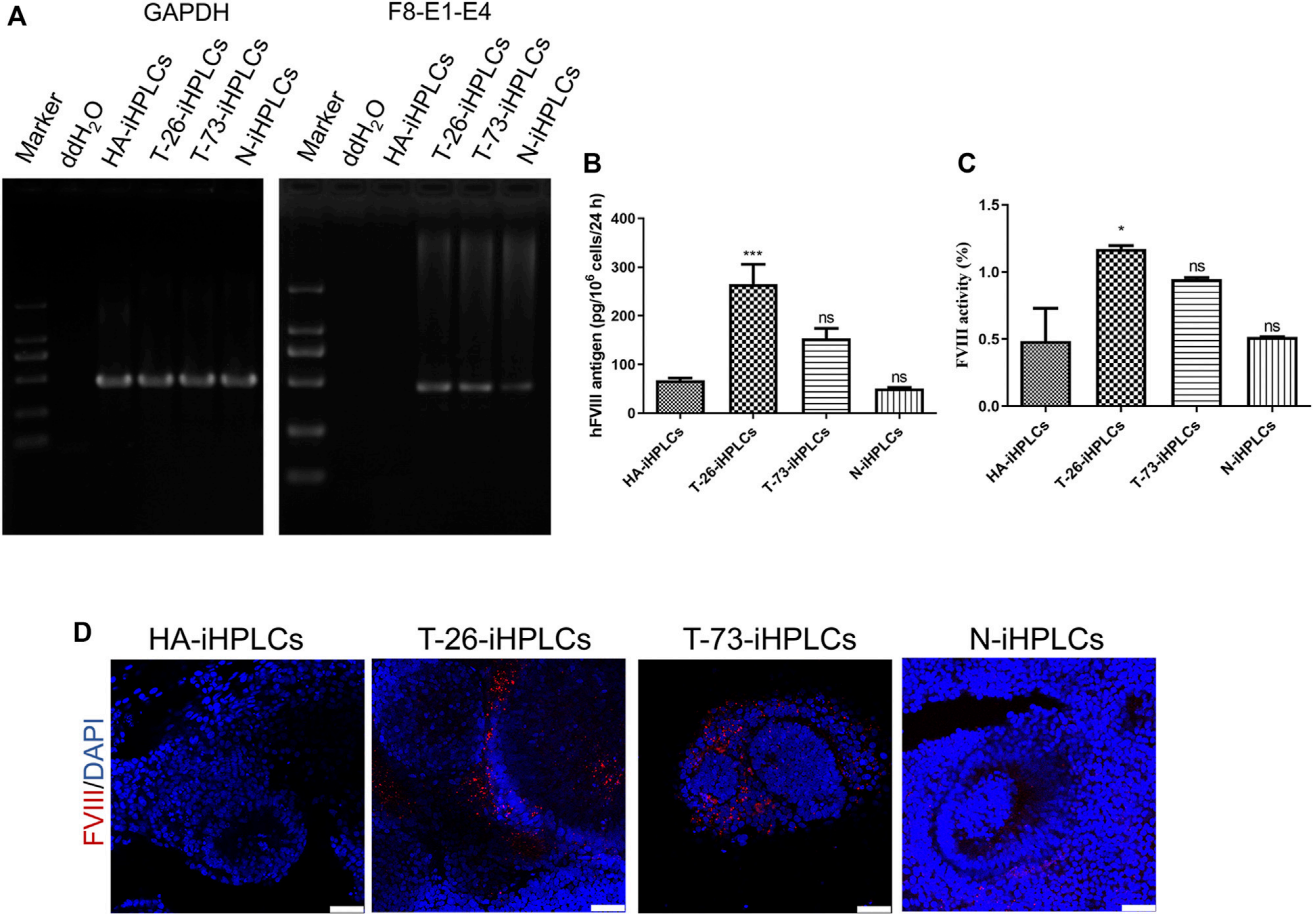
*F8* expression in gene corrected iPSCs derived iHPLCs. **(A)** RT-PCR analysis of F8 transcription in iHPLCs derived from iPSCs. F8-E1-E4 using primers targeting exons one and four of F8. GAPDH was used as a loading control. **(B)** ELISA of the FVIII antigen in iHPLCs differentiated from iPSCs. ***p < 0.001, vs. HA-iHPLCs group, ns, not significant compared with the HA-iHPLCs group. Data represent the mean ± SEM (*n* = 3 independent cultures). **(C)** FVIII activity detection of the supernatant from the iHPLCs. *p < 0.05, vs. HA-iHPLCs group, ns, not significant compared with the HA-iHPLCs group. Data represent the mean ± SEM (*n* = 3 independent cultures). **(D)** Immunofluorescence staining of FVIII (red) in iHPLCs, DAPI was used for nuclear staining. Scale bar: 50 µm.

## 3 Discussion

Considering the regulatory elements of the *F8* gene can be retained to the greatest extent, the strategy of *in situ* gene correction for HA was designed and performed. Wu *et al.* inserted the coding sequences of exon 23–26 into the exon 22-intron 22 junctions to correct the intron 22 inversion mutation. *F8* expression was restored in mesenchymal stem cells (MSCs) and endothelial cells (ECs) differentiated from the gene-corrected iPSCs (Wu et al., 2016). The targeting efficiency was 62.5% with the Neo selection cassette. Park *et al.* reverted the *F8* intron one or intron 22 inversion mutations in HA patient derived iPSCs using CRISPR/Cas9, with a frequency of 6.7% (Park et al., 2015). The ECs derived from the gene corrected iPSCs were transplanted into the HA mice and functionally rescued the FVIII deficiency. We previously deleted the coding sequences of B domain in *F8* gene precisely to rescue the HA with pathogenic mutations in B domain of *F8*. The *F8* expression and secretion were validated in the ECs derived from the B domain deleted iPSCs *in vitro* and *in vivo* (Hu et al., 2019; Hu et al., 2022). In this study, we firstly corrected the Inv1 mutation of *F8 via in situ* gene addition strategy in HA-iPSCs with an efficiency up to 10.19% without any screening.

This work suggests a feasible therapeutic gene addition strategy for HA involving large sequence variants. Luo *et al.* reported the *F8* expression and FVIII deficiency were rescued *via* injection of the AAV carrying CRISPR/SaCas9 and the donor plasmid with a promoter and the coding sequence of exon one into the HA mice with deletion of the promoter region and exon one of *F8* (Luo et al., 2021). However, the strategy wasn’t validated in cells or HA mice with Inv1 of *F8*. Here we firstly rescued the 140 kb reversion mutation by gene addition a 975 bp fragment in the HA-iPSCs with Inv1 mutation, providing a promising gene correction strategy for other genetic birth defects with large sequence variants.

Researchers have made many attempts in CRISPR-based strategies for targeted gene correction and made tremendous advances. The error-prone NHEJ repair pathway is the main DNA repair pathway in non-dividing cells and occurs during the whole cell cycle, whereas the HDR only occurs in S/G2 phase in dividing cells (Lau et al., 2020). The HMEJ strategy is based on both the targeted genomic site and the donor vector with homology arms flanking the recognition sequence of CRISPR/Cas9 cleaved *via* CRISPR/Cas9. It has been reported that a higher site-specific gene integration efficiency can be achieved by HMEJ based strategy than the classic HDR strategy. In this study, the NHEJ, HDR, and HMEJ based donor plasmid were designed and constructed. Consistent with previous reports, the gene integration efficiency was 10.19% in HMEJ group, higher than that in HDR group (6.25%) and NHEJ group (0.99%) without any drug screening.

Although the FVIII is mainly synthesized in liver sinusoidal endothelial cells (LSECs) under physiological conditions (Shahani et al., 2014; Hayakawa et al., 2021), many studies demonstrated that the ectopic expression of FVIII in hepatocytes was efficient and the FVIII deficiency in HA mice and HA patients were rescued (Bunting et al., 2018; Chen et al., 2019; Zhang et al., 2019). Successful amelioration of hemophilia A has been reported by several groups targeting the liver-expressed mouse *Alb* locus *in vivo* using AAV vectors to transfer the transgene into the liver (Sharma et al., 2015; Chen et al., 2019; Zhang et al., 2019). By integrating the promoter-less BDD-F8 into this locus, the transgene is expressed under the robust *Alb* promoter, achieving therapeutic levels of plasma FVIII for up to 7 months after injection. Conversely, this study integrated a foreign promoter to the endogenous *F8* coding sequence, using smaller integrated fragments (<1 kb) to repair *F8* compared to the more extensive BDD-F8 (over 4 kb) in previous studies. Our data demonstrated that FVIII expression and secretion were rescued in hepatocytes derived from gene corrected iPSCs. The iPSCs with hAAT-promoted *F8* cassette provide an adequate cell source for therapeutic hepatocytes. As the endogenous endothelial-expressed *F8* promoter was lost in cells of HA patient with Inv1 mutation during the chromosomal reversion and the integrated hAAT promoter is liver-specific, we did not differentiate the gene corrected iPSCs into LSECs as a control in this study. In our further studies, we will integrate an endothelial-specific promoter to correct the iPSCs and differentiate them into LSECs, and the comparison of FVIII expression in HPLCs and LSECs will be investigated.

In summary, we performed a CRISPR/Cas9 mediated HMEJ in HA-iPSCs with Inv1 by targeting gene addition of the hAAT promoter and *F8* exon one at the intron 1 with a high efficiency up to 10.19%. Both *F8* transcription and FVIII secretion were rescued in the hepatocytes derived from gene corrected iPSCs. This is the first report of an efficient *in situ* genetic addition strategy in HA-iPSCs with Inv1 mutation, while further *in vivo* experiments need to perform to evaluate the effectiveness. Hopefully, our findings suggest a feasible and promising *in situ* genetic addition strategy for HA involving large sequence variants.

## 4 Materials and methods

### 4.1 Characterization of iPSCs

The expression of iPSC surface markers were detected using immunofluorescence staining. iPS cells were fixed with 4% paraformaldehyde for 15 min, then permeabilized with DPBS (Thermo Fisher Scientific #C14190500BT, Waltham, MA, United States) contained 0.1% Triton-X100 for 15 min, followed by blocking with 5% bovine serum albumin (BSA, Geneview #FA016, St. Galveston, TX, United States) in DPBS for 30 min, then the cells were incubated with primary antibody diluted 1:100 in 5% bovine serum albumin in DPBS (OCT4 (Abcam #ab181557, Cambridge, United Kingdom), NANOG (Abcam #ab109250), stage-specific embryonic antigen (SSEA)-1 and SSEA-4 (Merck Millipore #SCR001, Billerica, MA, United States) at room temperature for 1 h. After washing with DPBS appropriately, cells were blocked again for 30 min and treated with secondary antibodies for 1 h in the dark. The nuclear staining was performed using 4′, 6′-diamidino-2-phenylindole (DAPI) (Thermo Fisher Scientific #D1306). Then the cells were photographed *via* fluorescence microscope (Leica DM IRB, Wetzlar, Germany).

For teratoma formation, iPSCs plated on 60 mm dish were digested and resuspended in 140 μL mTeSR1 (STEMCELL Technologies #85850, Vancouver, BC, Canada) with 70 μL Matrigel (Corning #354277, NY, United States). The iPSCs were transplanted into the groins of NSG mice subcutaneously. Eight weeks later, the formed teratoma was harvested, fixed, paraffin embedded, sectioned, stained with hematoxylin and eosin, photographed and analyzed. The care and use of the animals are in accordance with the guidelines of the Ethics Committee of the School of Life Sciences of Central South University. All operations involving animal experiments were approved by the Institutional Animal Care and Use Committee of School of Life Sciences of Central South University (No. 2021-2–12, Date: 12 March 2021).

### 4.2 Karyotype analysis of iPSCs

G-banding analysis of chromosomes was performed. The cells were incubated with 0.1 μg/mL colcemid (Sigma-Aldrich #D7385, St. Louis, MO, United States) for 4 h, followed by trypsinization, and hypotonic treatment with 0.075 M KCl for 10 min at 37°C. Then the cells were fixed with Carnoy fixative, and the metaphase chromosomes were spreaded using an air-drying method, then treated with Giemsa (Sigma-Aldrich #48900) and analyzed.

### 4.3 RNA-guided endonucleases (RGENs) design and plasmid construction

All short guide RNA oligos which were designed *via* CRISPOR (http://crispor.tefor.net/) were synthesized by Sangon Biotech. The plasmid pX601 (pX601-AAV-CMV:NLS-SaCas9-NLS-3xHA-bGHpA;U6:BsaI-sgRNA) was a gift from Feng Zhang (Addgene plasmid #61591; http://n2t.net/addgene:61591; RRID:Addgene_61591, Watertown, MA, United States) (Ran et al., 2015). The annealed complementary sgRNA oligos were ligated with the pX601 digested with BbsI (New England Biolabs #R3539, Ipswich, MA, United States) using T4 DNA ligase (Thermo Fisher Scientific #EL0011). To evaluate the activity, HEK293T cells were transfected with CRISPR/SaCas9 expression plasmid using Lipofectamine 2000 (Invitrogen #11668–019, Carlsbad, CA, United States). After 2 days, genomic DNA was extracted. The PCR products encompassing the targeted locus were purified and treated with mismatch-sensitive T7E1 (Vazyme #EN303-01, Nanjing, China). After electrophoresis, gene disruption was evaluated *via* a gel imaging system.

The donor plasmids NHEJ, HDR, HMEJ were synthesized by Sangon Biotech and were confirmed by Sanger sequencing.

### 4.4 Gene targeting

The iPSCs cultured on Matrigel were transfected with the human stem cell Nucleofector kit 2 (Lonza #VPH-5022, Alpharetta, GA, United States) using Nucleofector II (Lonza) set at program B016. 2.5 µg of CRISPR/Cas9 plasmid and the donor plasmid were used to transfect 1×10^6^ cells (HA-iPSCs). After 2 days, 1,000 single transfected cells were seeded on Matrigel coated 6-cm dish in CloneR medium (STEMCELL Technologies #1000691). Approximately 12 days later, clones were picked up and identified by PCR and Sanger sequencing. PCR was performed using two pairs of primers: F8-5F, 5′- CAA​AAT​GAT​ACA​GAA​AGT​AGA​ATG​G-3′ and F8-5R, 5′- CAG​GGA​GGG​CTG​TGT​GTT​T-3'; and F8-3F, 5′- TTT​CTG​AGC​CAG​GTA​CAA​TGA-3′ and F8-3R, 5′- GCT​GTA​ATT​CAG​AAT​CAG​TCC​TAC-3'. The PCR products were sequenced by Sanger sequencing. The N-iPSCs (DYR0100) purchased from ATCC were used as normal control.

### 4.5 Analysis of potential off-target sites

The potential off-target sites of sgRNA4 were searched using CHOPCHOP (http://chopchop.cbu.uib.no/) (Montague et al., 2014), seven potential sites were predicted for mismatches of up to four nucleotides. Then the regions encompassing the seven potential sites in gene-edited clones were PCR amplified, followed by Sanger sequencing (Supplementary Table S1). Different indels in HA-iPSCs and genetically edited iPSCs were used to evaluate off-target effects. Primer sequences are shown in Supplementary Table S2.

### 4.6 RT-PCR

Total RNA isolated with TRIzol reagent (Sigma-Aldrich #T9424) was digested with DNase for 30 min, followed by reverse transcribed *via* HiScript II 1st Strand cDNA Synthesis Kit (Vazyme #R212). The primers were based on exons one and four to detect the *F8* transcripts. Glyceraldehyde-3-phosphate dehydrogenase (GAPDH) was amplified to represent an endogenous control. Primer sequences are shown in Supplementary Table S3.

### 4.7 Differentiation into hepatocytes from human iPSCs

Derivation of hepatocyte from human iPSCs was performed based on Yukiko Toba’s protocol with some modifications (Labun et al., 2019). Briefly, for induction of the definitive endoderm cells, iPSCs were cultured in Differ1 medium (RPMI1640 medium, Hyclone #SH30027, South Logan, UT, United States; 100 ng/mL Activin A, Peprotech #120–14P, Rocky, Hill, NJ, United States; 1 × GlutaMAX, Thermo Fisher Scientific #35050061; 0.2% fetal bovine serum, Thermo Fisher Scientific #16000044, 1 × B27 Supplement Minus Vitamin A, Thermo Fisher Scientific #12587010) for 4 days.

For the hepatoblast-like cells differentiation, the definitive endoderm cells were cultured in Differ2 medium (RPMI1640 medium, 20 ng/mL recombinant human BMP4 (Biolegend #595202, San Diego, CA, United States); 1 × GlutaMAX, and 1 × B27 Supplement Minus Vitamin A) for 5 days.

To perform the hepatocyte differentiation, the medium was replaced with Diff3 medium (RPMI1640 medium, 20 ng/mL HGF, Peprotech #100-39H; 1 × GlutaMAX, and 1 × B27 Supplement Minus Vitamin A) and cultured for 5 days. Then the cells were cultured for 11 days in Hepatocyte Culture Medium (HCM, Lonza #cc3198) containing 20 ng/mL Recombinant Human Oncostatin M (OsM, Peprotech #300–10).

### 4.8 Characterization of hepatocytes

Immunofluorescence staining of hepatocyte was performed as that described for iPSC characterization. The primary antibodies used were anti-SOX17 (R&D SYSTERM #AF 1924, Minneapolis, MN, United States), anti-FOXA2 (Merck Millipore #07–633), anti-FVIII N-terminus antibody (Santa Cruz Biotechnology #sc27649, Dallas, TX, United States), and anti-AFP (Sigma-Aldrich #A8452), anti-ALB (R&D SYSTERM #MAB1455).

For periodic acid schiff stain, the hepatocytes on Day 25 were stained with the Periodic Acid Schiff Stain Kit (Solarbio #G1280, Beijing, China) according to the manufacturer’s instructions. The periodic acid schiff stain was detected by microscopy.

For indocyanine green (ICG) uptake assay, hepatocytes on Day 25 were treated with 1 mg/mL ICG (Sigma-Aldrich #1340009) for 30 min, then washed with DPBS thoroughly and cultured in fresh Hepatocyte Culture Medium. The cells were detected by microscopy. After 12 h, the cells were observed using microscopy.

### 4.9 FVIII ELISA

Culture supernatants of mature hepatocytes from 12-well plates were harvested in triplicate after medium replacement for 24 h. ELISA was performed with paired antibodies for ELISA-Factor VIII:C (Cedarlane #CL20035K, Burlington, ON, Canada) according to manufacturer instructions. The standard curves were constructed using serial dilutions of normal pooled plasma, with a correlation coefficient (R2) greater than 0.990 using a semilog fit.

### 4.10 FVIII activity assay

For the FVIII activity assay, 24-hour-old culture supernatants of mature hepatocytes were collected. The activated partial thromboplastin time (aPTT) was detected using a Destiny Max hemostasis analyzer (Tcoag, Lemgo, Germany) according to the manufacturer’s instructions.

### 4.11 Statistical analysis

GraphPad Prism 8.0 was used for data analysis. Data were analyzed using ANOVA for more than two groups.

## Data Availability

The original contributions presented in the study are included in the article/Supplementary Material, further inquiries can be directed to the corresponding authors.

## Supplementary material

SUPPLEMENTARY FIGURE S1 Characterization of HA-iPSCs. **(A)** Immunofluorescence staining of HA-iPSCs and the results indicated HA-iPSCs expressed the markers NANOG, OCT4, SSEA-4 but not SSEA-1. DAPI was used for nuclear staining. Scale bar: 50 µm. **(B)** H&E staining of teratomas derived from HA-iPSCs. The teratomas contained three germ layers (ectoderm, mesoderm and endoderm). Scale bar: 200 µm. **(C)** Karyotype of HA-iPSCs.

SUPPLEMENTARY FIGURE S2 Sanger sequencing of potential off-target sites in T-26-iPSCs and T-73-iPSCs using F8-sg4. No indels were found at the sites.

SUPPLEMENTARY TABLE S1 Potential off-target sites of sg4 predicted by the CHOPCHOP.

SUPPLEMENTARY TABLE S2 Primers used in sg4 off-target analysis.

SUPPLEMENTARY TABLE S3 Primers used in RT-PCR detection.

## References

[B1] Batty P. Lillicrap D. (2019). Advances and challenges for hemophilia gene therapy. Hum. Mol. Genet. 28 (1), R95–R101. 10.1093/hmg/ddz157 31332444

[B2] Berntorp E. Fischer K. Hart D. P. Mancuso M. E. Stephensen D. Shapiro A. D. (2021). Haemophilia. Nat. Rev. Dis. Prim. 7 (1), 45. 10.1038/s41572-021-00278-x 34168126

[B3] Bunting S. Zhang L. Xie L. Bullens S. Mahimkar R. Fong S. (2018). Gene therapy with BMN 270 results in therapeutic levels of FVIII in mice and primates and normalization of bleeding in hemophilic mice. Mol. Ther. 26 (2), 496–509. 10.1016/j.ymthe.2017.12.009 29292164 PMC5835117

[B4] Chen H. Shi M. Gilam A. Zheng Q. Zhang Y. Afrikanova I. (2019). Hemophilia A ameliorated in mice by CRISPR-based *in vivo* genome editing of human Factor VIII. Sci. Rep. 9(1), 16838. 10.1038/s41598-019-53198-y 31727959 PMC6856096

[B5] Fahiminiya S. Rivard G. E. Scott P. Montpetit A. Bacot F. St-Louis J. (2021). A full molecular picture of F8 intron 1 inversion created with optical genome mapping. Haemophilia 27 (5), e638–e640. 10.1111/hae.14375 34232555

[B6] Hayakawa M. Sakata A. Hayakawa H. Matsumoto H. Hiramoto T. Kashiwakura Y. (2021). Characterization and visualization of murine coagulation factor VIII-producing cells *in vivo* . Sci. Rep. 11 (1), 14824. 10.1038/s41598-021-94307-0 34290295 PMC8295325

[B7] Hu Z. Hu X. Pang J. Wang X. Lin Peng S. Li Z. (2015). Establishment of hemophilia A patient-specific inducible pluripotent stem cells with urine cells. Zhonghua Yi Xue Yi Chuan Xue Za Zhi 32 (5), 609–614. 10.3760/cma.j.issn.1003-9406.2015.05.001 26418976

[B8] Hu Z. Li Z. Wu Y. Zhao J. Wu L. Zhou M. (2022). Targeted B-domain deletion restores F8 function in human endothelial cells and mice. Signal Transduct. Target Ther. 7 (1), 189. 10.1038/s41392-022-01016-9 35718817 PMC9207027

[B9] Hu Z. Zhou M. Wu Y. Li Z. Liu X. Wu L. (2019). ssODN-mediated in-frame deletion with CRISPR/Cas9 restores FVIII function in hemophilia A-patient-derived iPSCs and ECs. Mol. Ther. Nucleic Acids 17, 198–209. 10.1016/j.omtn.2019.05.019 31261034 PMC6610636

[B10] Labun K. Montague T. G. Krause M. Torres Cleuren Y. N. Tjeldnes H. Valen E. (2019). CHOPCHOP v3: Expanding the CRISPR web toolbox beyond genome editing. Nucleic Acids Res. 47 (1), W171–W174. 10.1093/nar/gkz365 31106371 PMC6602426

[B11] Lassalle F. Jourdy Y. Jouan L. Swystun L. Gauthier J. Zawadzki C. (2020). The challenge of genetically unresolved haemophilia A patients: Interest of the combination of whole F8 gene sequencing and functional assays. Haemophilia 26 (6), 1056–1063. 10.1111/hae.14179 33094873

[B12] Lau C. H. Tin C. Suh Y. (2020). CRISPR-based strategies for targeted transgene knock-in and gene correction. Fac. Rev. 9, 20. 10.12703/r/9-20 33659952 PMC7886068

[B13] Li M. Tang X. You W. Wang Y. Chen Y. Liu Y. (2021). HMEJ-mediated site-specific integration of a myostatin inhibitor increases skeletal muscle mass in porcine. Mol. Ther. Nucleic Acids 26, 49–62. 10.1016/j.omtn.2021.06.011 34513293 PMC8411015

[B14] Luo S. Li Z. Dai X. Zhang R. Liang Z. Li W. (2021). CRISPR/Cas9-Mediated *in vivo* genetic correction in a mouse model of hemophilia A. Front. Cell Dev. Biol. 9, 672564. 10.3389/fcell.2021.672564 34485274 PMC8415270

[B15] Marrone L. Marchi P. M. Azzouz M. (2022). Circumventing the packaging limit of AAV-mediated gene replacement therapy for neurological disorders. Expert Opin. Biol. Ther. 22 (9), 1163–1176. 10.1080/14712598.2022.2012148 34904932

[B16] Montague T. G. Cruz J. M. Gagnon J. A. Church G. M. Valen E. (2014). CHOPCHOP: A CRISPR/Cas9 and TALEN web tool for genome editing. Nucleic Acids Res. 42, W401–W407. Web Server issue). 10.1093/nar/gku410 24861617 PMC4086086

[B17] Ozelo M. C. Mahlangu J. Pasi K. J. Giermasz A. Leavitt A. D. Laffan M. (2022). Valoctocogene roxaparvovec gene therapy for hemophilia A. N. Engl. J. Med. 386 (11), 1013–1025. 10.1056/NEJMoa2113708 35294811

[B18] Park C. Y. Kim D. H. Son J. S. Sung J. J. Lee J. Bae S. (2015). Functional correction of large factor VIII gene chromosomal inversions in hemophilia A patient-derived iPSCs using CRISPR-cas9. Cell Stem Cell 17 (2), 213–220. 10.1016/j.stem.2015.07.001 26212079

[B19] Pasi K. J. Rangarajan S. Mitchell N. Lester W. Symington E. Madan B. (2020). Multiyear follow-up of AAV5-hFVIII-SQ gene therapy for hemophilia A. N. Engl. J. Med. 382(1), 29–40. 10.1056/NEJMoa1908490 31893514

[B20] Perrin G. Q. Herzog R. W. Markusic D. M. (2019). Update on clinical gene therapy for hemophilia. Blood 133 (5), 407–414. 10.1182/blood-2018-07-820720 30559260 PMC6356985

[B21] Ragni M. V. (2021). Hemophilia as a blueprint for gene therapy. Science 374 (6563), 40–41. 10.1126/science.abg0856 34591611

[B22] Ran F. A. Cong L. Yan W. X. Scott D. A. Gootenberg J. S. Kriz A. J. (2015). *In vivo* genome editing using *Staphylococcus aureus* Cas9. Nature 520 (7546), 186–191. 10.1038/nature14299 25830891 PMC4393360

[B23] Rodriguez-Merchan E. C. De Pablo-Moreno J. A. Liras A. (2021). Gene therapy in hemophilia: Recent advances. Int. J. Mol. Sci. 22 (14), 7647. 10.3390/ijms22147647 34299267 PMC8306493

[B24] Shahani T. Covens K. Lavend'homme R. Jazouli N. Sokal E. Peerlinck K. (2014). Human liver sinusoidal endothelial cells but not hepatocytes contain factor VIII. J. Thromb. Haemost. 12 (1), 36–42. 10.1111/jth.12412 24118899

[B25] Sharma R. Anguela X. M. Doyon Y. Wechsler T. DeKelver R. C. Sproul S. (2015). *In vivo* genome editing of the albumin locus as a platform for protein replacement therapy. Blood 126 (15), 1777–1784. 10.1182/blood-2014-12-615492 26297739 PMC4600017

[B26] Song X. Zhong J. Xue F. Chen L. Li H. Yuan D. (2021). An overview of patients with haemophilia A in China: Epidemiology, disease severity and treatment strategies. Haemophilia 27(1), e51-e59. 10.1111/hae.14217 33245829

[B27] Tornabene P. Trapani I. (2020). Can adeno-associated viral vectors deliver effectively large genes? Hum. Gene Ther. 31(1-2), 47–56. 10.1089/hum.2019.220 31916856

[B28] Verdera H. C. Kuranda K. Mingozzi F. (2020). AAV vector immunogenicity in humans: A long journey to successful gene transfer. Mol. Ther. 28 (3), 723–746. 10.1016/j.ymthe.2019.12.010 31972133 PMC7054726

[B29] Wu Y. Hu Z. Li Z. Pang J. Feng M. Hu X. (2016). *In situ* genetic correction of F8 intron 22 inversion in hemophilia A patient-specific iPSCs. Sci. Rep. 6, 18865. 10.1038/srep18865 26743572 PMC4705535

[B30] Yao X. Wang X. Hu X. Liu Z. Liu J. Zhou H. (2017). Homology-mediated end joining-based targeted integration using CRISPR/Cas9. Cell Res. 27 (6), 801–814. 10.1038/cr.2017.76 28524166 PMC5518881

[B31] Yuan M. Zhang J. Gao Y. Yuan Z. Zhu Z. Wei Y. (2021). HMEJ-based safe-harbor genome editing enables efficient generation of cattle with increased resistance to tuberculosis. J. Biol. Chem. 296, 100497. 10.1016/j.jbc.2021.100497 33675752 PMC8038940

[B32] Zhang J. P. Cheng X. X. Zhao M. Li G. H. Xu J. Zhang F. (2019). Curing hemophilia A by NHEJ-mediated ectopic F8 insertion in the mouse. Genome Biol. 20(1), 276. 10.1186/s13059-019-1907-9 31843008 PMC6912951

