# Supplementary material for: Correction of F8 intron 1 inversion in hemophilia A patient-specific iPSCs by CRISPR/Cas9 mediated gene editing
Source: Front Genet. 2023 Mar 9;14:1115831. doi: 10.3389/fgene.2023.1115831 (PMC10033665; doi:10.3389/fgene.2023.1115831)
Supplement: Supplementary file 1 [file Table1.DOCX]

Supplementary Material

FVIII restoration via targeted gene rescue in HA-patient specific iPSCs and hepatocyte-like cells

Zhiqing Hu ^1,^†, Yong Wu ^1,^†, Rou Xiao ^1^, Junya Zhao ^1^, Yan Chen ^1^, Lingqian Wu ^1^, Miaojin Zhou ^1,^*, and Desheng Liang ^1,^*

*** Correspondence:** Corresponding Author: Miaojin Zhou, [zhoumiaojin@sklmg.edu.cn](mailto:zhoumiaojin@sklmg.edu.cn); Desheng Liang, liangdesheng@sklmg.edu.cn

## Supplementary Figures


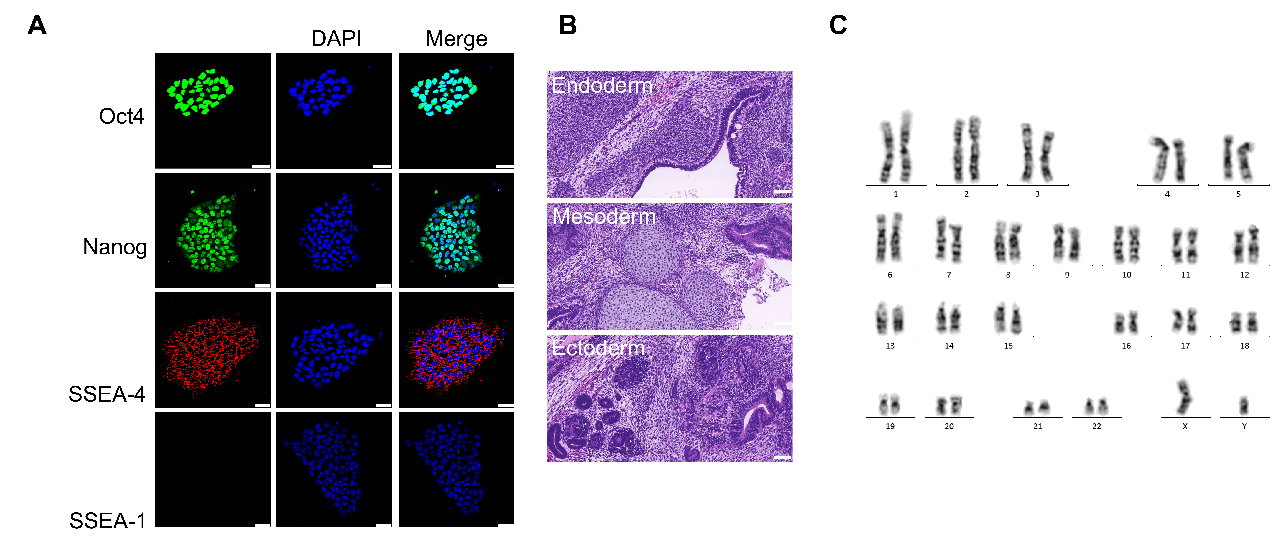


**Supplementary Figure 1** Characterization of HA-iPSCs. (A) Immunofluorescence staining of HA-iPSCs and the results indicated HA-iPSCs expressed the markers NANOG, OCT4, SSEA-4 but not SSEA-1. DAPI was used for nuclear staining. Scale bar: 50 µm. (B) H&E staining of teratomas derived from HA-iPSCs. The teratomas contained three germ layers (ectoderm, mesoderm and endoderm). Scale bar: 200 µm. (C) Karyotype of HA-iPSCs.

**
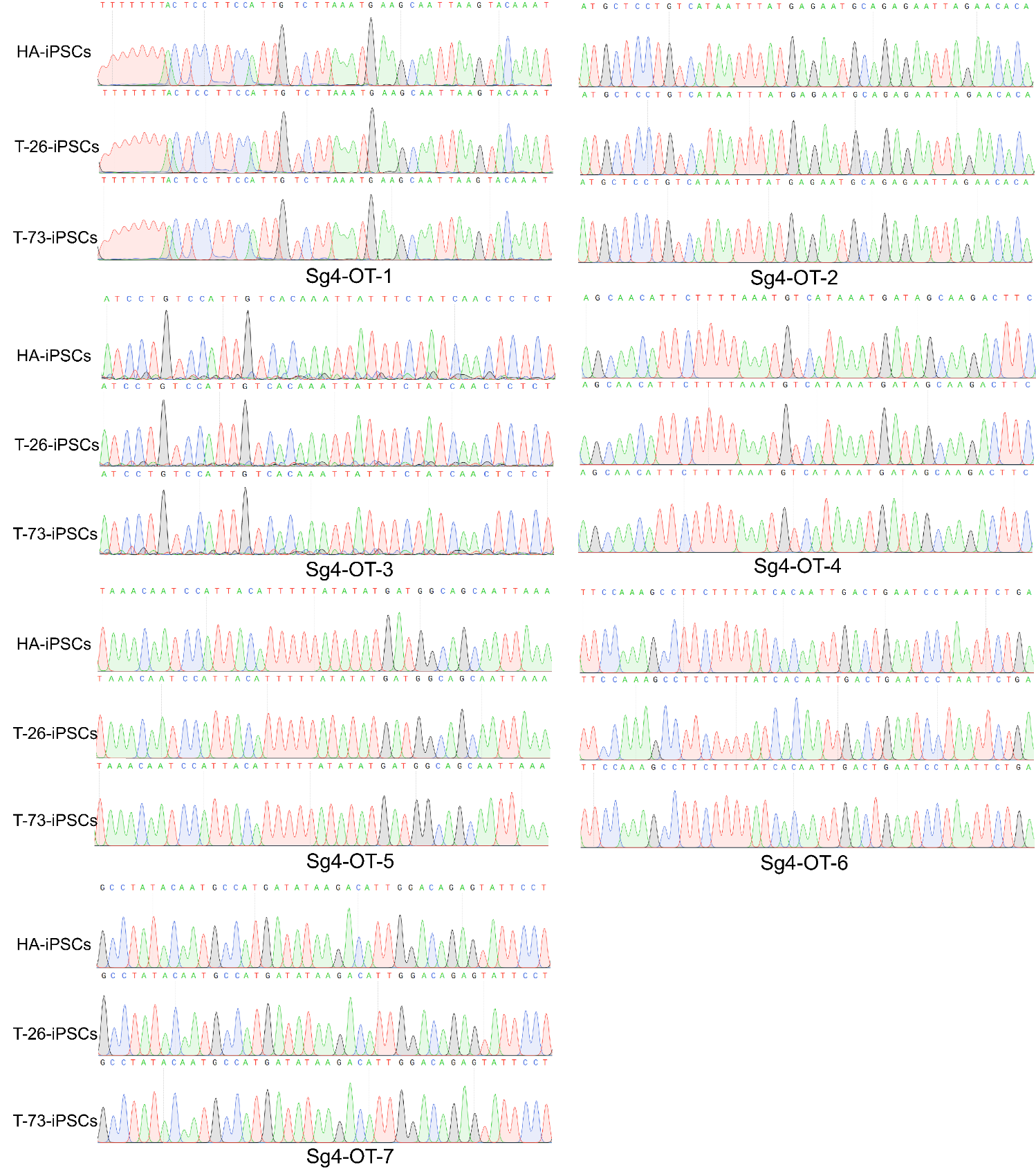
 Supplementary Figure 2** Sanger sequencing of potential off-target sites in T-26-iPSCs and T-73-iPSCs using F8-sg4. No indels were found at the sites.

## Supplementary Tables

Supplementary Table 1. Potential off-target sites of sg4 predicted by the CHOPCHOP

| **sequence** | **mismatches** | **locus** |
| --- | --- | --- |
| TGCTTCATTTAAGACAATGGAAGGAGT | 4MMs[1:2:4:12] with sg4 | chr5:28143991 |
| GTCATAATTTATGAGAATGCAGAGAAT | 4MMs[2:6:15:20] with sg4 | chr4:130003151 |
| GAAATAATTTGTGACAATGGACAGGAT | 4MMs[2:3:6:11] with sg4 | chr9:20033513 |
| GCTATCATTTATGACATTTAAAAGAAT | 4MMs[3:17:19:20] with sg4 | chr6:48318877 |
| GCCATCATATATAAAAATGTAATGGAT | 4MMs[9:13:15:20] with sg4 | chr6:114946237 |
| GCCTTCTTTTATCACAATTGACTGAAT | 4MMs[4:7:13:19]with sg4 | chr6:168677105 |
| GCCATGATATAAGACATTGGACAGAGT | 4MMs[6:9:12:17] with sg4 | chr3:139816821 |

The off-target sites were predicted by the CHOPCHOP. The mismatch is indicated by red letters.

Supplementary Table 2. Primers used in sg4 off-target analysis

| **Site** | **Forward primer** | **Reverse primer** | **Expected product size, bp** |
| --- | --- | --- | --- |
| Sg4-OT-1 | TGACAGTTCAGCTTCCTCCG | CTATGTCCTATGTCAAAGCTTTTCA | 124 |
| Sg4-OT-2 | GAGAGTCACCACTAATCTACGAGCC | AAACTTGGCTTACAACTTTGATTCC | 442 |
| Sg4-OT-3 | AGATTTTCAGGGTTTAGAAATCTGC | GTCAGGGCACTGGTGGGAG | 605 |
| Sg4-OT-4 | TCCTTATGGGTATCTACAATCCTTT | ACACAAGAAATTAATCTGGCAATTT | 294 |
| Sg4-OT-5 | GTTTCTCTCTTCAAGGCTGTATCAC | CCTTCCTAAGCGATGCCAA | 381 |
| Sg4-OT-6 | GACATTTTCCTTTAGCTCAGAGAGG | CTTGAGTCCTCTTCGTGGTTTTA | 396 |
| Sg4-OT-7 | TTTATTGTCACAGGTGTTTGGAGTT | GTTTGCTTTGTGGCATTCTTGA | 419 |

Supplementary Table 3. Primers used in RT-PCR detection

| **Primer name** | **Primer sequence (5’-3’)** |
| --- | --- |
| GAPDH-F | GGGGAGCCAAAAGGGTCATCATCT |
| GAPDH-R | GACGCCTGCTTCACCACCTTCTTG |
| F8-E1 | GCCTTTTGCGATTCTGCTTTA |
| F8-E4 | ATTGGACCATTCTCTTTCAGGAC |
| hAAT-E5 | GGGGAAACTACAGCACCTGGAA |
| hAAT-E7 | TCAGTCCCTTTCTCGTCGATGGT |
